# Lipid Droplet-Associated Hydrolase Promotes Lipid Droplet Fusion and Enhances ATGL Degradation and Triglyceride Accumulation

**DOI:** 10.1038/s41598-017-02963-y

**Published:** 2017-06-02

**Authors:** Young-Hwa Goo, Se-Hee Son, Antoni Paul

**Affiliations:** Department of Molecular and Cellular Physiology, Albany Medical College, Albany, NY USA

## Abstract

Lipid droplet (LD)-associated hydrolase (LDAH) is a newly identified LD protein abundantly expressed in tissues that predominantly store triacylglycerol (TAG). However, how LDAH regulates TAG metabolism remains unknown. We found that upon oleic acid loading LDAH translocalizes from the ER to newly formed LDs, and induces LD coalescence in a tubulin-dependent manner. LDAH overexpression and downregulation in HEK293 cells increase and decrease, respectively, TAG levels. Pulse and chase experiments show that LDAH enhances TAG biogenesis, but also decreases TAG turnover and fatty acid release from cells. Mutations in predicted catalytic and acyltransferase motifs do not influence TAG levels, suggesting that the effect is independent of LDAH’s enzymatic activity. However, a LDAH alternative-splicing variant missing 90 amino acids at C-terminus does not promote LD fusion or TAG accumulation, while it still localizes to LDs. Interestingly, LDAH enhances polyubiquitination and proteasomal degradation of adipose triglyceride lipase (ATGL), a rate limiting enzyme of TAG hydrolysis. Co-expression of ATGL reverses the changes in LD phenotype induced by LDAH, and both proteins counterbalance their effects on TAG stores. Together, these studies support that under conditions of TAG storage in LDs LDAH plays a primarily lipogenic role, inducing LD growth and enhancing degradation of ATGL.

## Introduction

Eukaryotic cells are able to store triacylglycerol (TAG) within cytosolic lipid droplets (LDs) to use it as a source of energy in times of scarcity. By isolating lipid from other cell components, LDs also buffer cytotoxic effects caused by excess of free fatty acids or free cholesterol^1, 2^. However, LDs are no longer considered passive lipid depots, and growing evidences indicate that they are quite dynamic organelles involved in multiple cellular and metabolic processes^2–4^. Despite their critical physiological functions, overabundance of LDs is a common feature in the pathogenesis of highly prevalent metabolic disorders such as obesity, diabetes, hepatic steatosis, and atherosclerosis^3, 4^. LDs are built of a monolayer of amphipathic lipids and LD-associated proteins (LDAPs), which stabilize a core of hydrophobic lipids within the aqueous cytosol^4–6^. With few exceptions, such as steroidogenic cells and foam cells in atherosclerotic lesions where cholesterol ester (CE) is more abundant, TAG is the main lipid species stored in the core of LDs. The final steps in TAG and CE synthesis primarily take place in the endoplasmic reticulum (ER). According to the prevalent model of LD biogenesis, LDs bud off the external layer of the ER membrane following progressive accumulation of neutral lipids between the leaflets^7–10^. How subsequent LD growth takes place still remains poorly understood. Different mechanisms that have been proposed to mediate the process include lipid transfer through ER-LD or LD-LD contact sites, *in situ* lipid synthesis, and LD fusion^11–13^.

The structure and metabolism of LDs are mainly regulated by LDAPs. LDAH is an evolutionarily conserved protein that has recently been identified in several proteomic analyses of purified LD fractions^14–17^. Like most lipases and esterases, LDAH is predicted to be built in a α/β hydrolase fold^15^. Its sequence harbors a highly conserved consensus GxSxG esterase/lipase catalytic motif, and conserved aspartate and hystidine residues could complete a functional catalytic triad. LDAH is highly expressed in macrophages, including foam cells within mouse and human atherosclerotic lesions^15^. Wild-type LDAH, but not a mutant lacking the active-site serine, displayed weak hydrolytic activity against cholesterol ester (CE)^15^. LDAH is also abundantly found in several tissues that predominantly store TAG, including liver and white and brown adipose tissues^15^. Intriguingly, in agreement with other groups we did not detect hydrolytic activity against TAG *in vitro*, but it was shown that LDAH is involved in LD clustering and fusion^14, 15^. Given that LDAH associates with TAG-rich LDs but does not seem to work as a TAG hydrolase, the aims of this study were to investigate its role in the biology of TAG-rich LDs and its impact on TAG homeostasis. The effects of the different LDAH splicing variants were compared, and mutants lacking the catalytic site as well as novel potential acyltransferase motifs we identified were also studied. The main conclusion of these studies is that in contrast to what its lipase-like structure suggested, under metabolic conditions that promote TAG storage in LDs LDAH plays a lipogenic role.

## Results

### LDAH translocates from the ER to nascent LDs, and increases LD size by promoting fusion in a tubulin-dependent fashion

**Figure 1:**
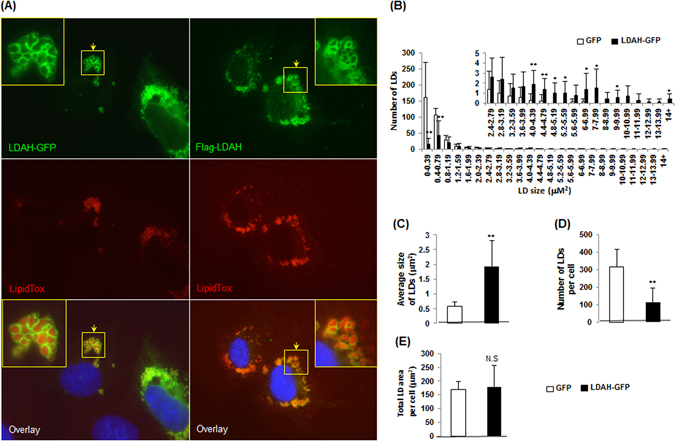
LDAH is a cytosolic protein that localizes to LDs upon OA loading. (**A**) LDAH-GFP (left) or Flag-tagged LDAH (right) were transfected to HeLa cells, and cells were treated with OA (360 μM for 24 h). Anti-Flag antibody (green) was used to detect Flag-LDAH, and LipidTox (red) was used to stain neural lipids. Nuclei were stained with DAPI (blue). Yellow arrows indicate LDAH associated with merged LDs. (**B**–**E**) Images of GFP or LDAH-GFP transfected cells loaded with OA were taken by fluorescence microscope and used for morphometric analysis of LDs to determine the effects of LDAH on LD size distribution (**B**); average size of LDs (**C**); average number of LDs per cell (**D**); and total LD area per cell (**E**). Ten cells per group were used for the measurements. *p < 0.05, **p < 0.005.

**Figure 2:**
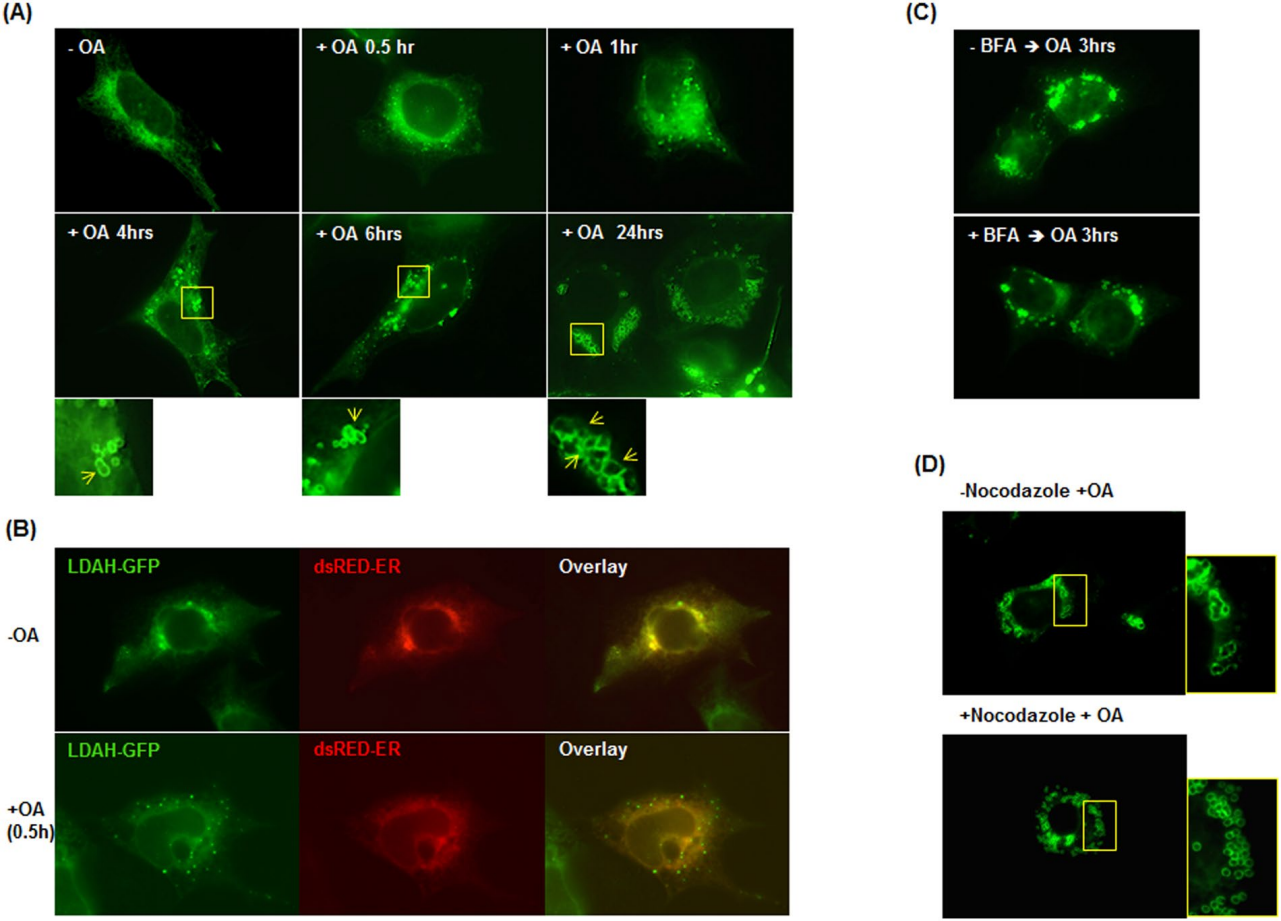
LDAH increases LD size by promoting fusion in a tubulin-dependent manner. (**A**) HEK293 cells were transfected with LDAH-GFP, and remained untreated or were incubated with 360 μM of OA for the indicated periods of time. Yellow arrows indicate fused LDs. (**B**) HEK293 cells were co-transfected with LDAH-GFP (green) and dsRed2-ER (red). Cells remained untreated or were treated with OA (360 μM) for 30 min. (**C**) LDAH-GFP overexpressing HEK293 cells remained untreated or were pre-treated with brefeldin A (BFA) for 30 min. OA (360 μM) was then added to the culture media for 3 h. LDAH’s localization to LDs was determined by fluorescence microscopy (green). **(D)** LDAH-GFP expressing HEK293 cells remained untreated or were pre-treated with nocodazole (2 μg/ml) for 30 min. OA (360 μM) was then added for 24 h.

To study LDAH’s role in TAG homeostasis, HeLa and HEK293 cells were treated with OA to generate TAG-rich LDs. As we previously observed in cholesterol-laden cells, in both cell types LDAH readily localized to the LD perimeter (Fig. 1A)^15^. However, a remarkable observation was the presence of large lipid bodies of unusual organic-looking morphology (Fig. 1A). Atypical LD morphology was obvious in cells transfected with either Flag- or GFP-tagged LDAH, and changes were more pronounced when higher doses of OA were used to enhance LD formation and growth (Fig. 1A and Figure S1). Morphometric studies confirmed that LDAH overexpression considerably increases the average LD size, while it decreases the total number of LDs (Fig. 1B–E). We next performed time-course experiments to study LDAH’s interaction with LDs in response to OA loading. In untreated cells, LDAH-GFP was detectable throughout the cytosol, but fluorescence was usually stronger in perinuclear regions (Fig. 2A). A similar pattern was observed in untreated cells transfected with Flag-LDAH (Figure S1). Discrete green fluorescent puncta were evident in the cytosol as early as 30 min after OA (360 μM) was added to the culture media, and after 1 h intense fluorescence decorated the perimeter of small circular LDs. At 4 h the fluorescent signal had already switched to a predominantly LD pattern, suggesting high affinity of LDAH for LDs from early stages of generation. Merged LDs were already seen at this time, and increased in number and size through the 24 h time point (Fig. 2A). The ER is the primary site of TAG and LD synthesis. In untreated cells and during early stages of LD formation, LDAH-GFP significantly overlapped with the ER marker dsRed, indicating that in the absence of LDs LDAH is in close association with the ER (Fig. 2B). The association of some LDAPs to LDs has been proposed to depend on the ER-Golgi transport machinery^18^. However, pretreatment with brefeldin A (BFA), a drug that interferes with the ER-Golgi transport, did not prevent LDAH’s association with LDs, suggesting that LDAH transfers to LDs directly from the cytosol or from the ER (Fig. 2C)^19^.

The morphological changes induced by LDAH are highly reminiscent of LD fusion. It was previously shown that LD fusion is dependent on microtubules, and that the process can be inhibited by treatment with nocodazole to disrupt tubulin polymerization^20, 21^. Thus, we asked whether nocodazole would also prevent the changes in LD morphology induced by LDAH. Immunofluorescence with an anti-tubulin antibody in LDAH-GFP overexpressing cells showed some overlap between tubulin and LDAH (Figure S2), but pretreatment with nocodazole did not prevent LDAH targeting to LDs at early (Figure S3) or late time points of OA loading (Fig. 2D), indicating that microtubules are not required for LDAH delivery to LDs. However, nocodazole prevented LD merging, and under nocodazole treatment LDAH-GFP outlined the perimeter of cytoplasmic LDs that, while still clustered, displayed a typical circular morphology (Fig. 2D). Thus, under basal conditions LDAH is in close association with the ER, but upon OA loading it rapidly associates with newly formed LDs, and promotes LD fusion in a process that is dependent of intact microtubules.

### LDAH increases intracellular TAG stores

**Figure 3:**
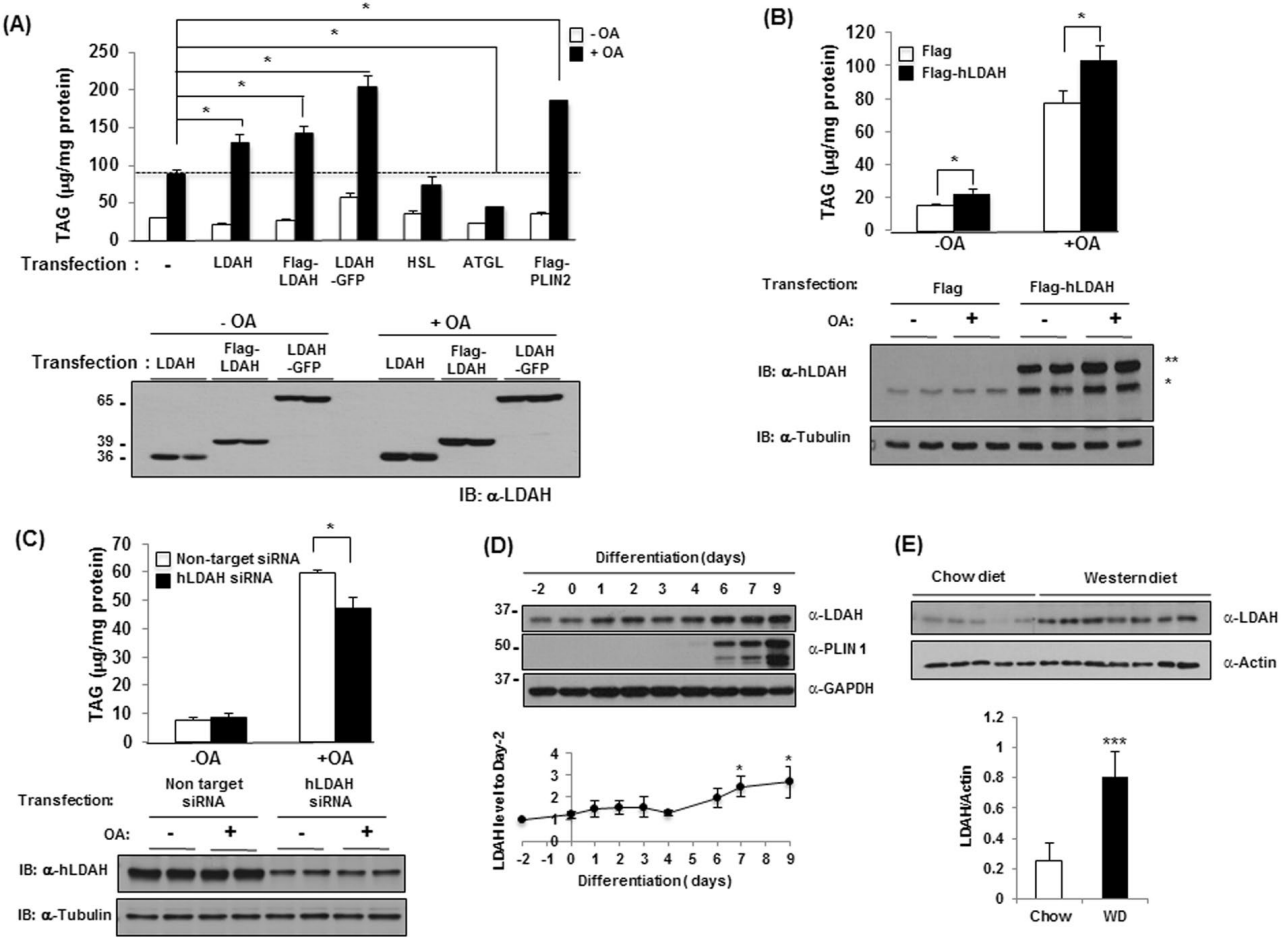
LDAH overexpression increases and LDAH downregulation decreases cellular TAG content. (**A**) Flag, LDAH, Flag-LDAH, LDAH-GFP, HSL, ATGL, or PLIN2 were transfected to HEK293 cells. After 24 h, cells were cultured with or without OA (360 μM) for 24 h. TAG content was measured biochemically and normalized to protein as described in materials and methods. Expression of LDAH with different tags was determined with anti-LDAH antibody (bottom). *p < 0.05. (**B**) Flag or Flag-tagged human LDAH (hLDAH) were transfected to HEK293 cells, and cells were cultured with or without OA (360 μM). Intracellular TAG levels were determined and normalized to protein. hLDAH was detected by immunoblotting with anti-hLDAH antibody. *; endogenous LDAH, **; Flag-hLDAH. **(C)** Non-target and human LDAH siRNAs were transfected to HEK293 cells. After 48 h, cells were treated with OA (360 μM) for 24 h, and cellular TAG levels were determined. The reduction in LDAH by siRNAs was confirmed by immunoblotting with anti-hLDAH antibody. (**D**) LDAH and PLIN1 expression during 3T3-L1 differentiation, and quantification of three independent experiments. *p < 0.05. **(E)** LDAH expression in white adipose tissues of mice fed either regular chow or Western diet (WD). LDAH was detected with anti-LDAH antibody. ***p < 0.001.

After establishing that LDAH promotes LD growth, we asked whether it would also impact intracellular TAG stores. As seen in Fig. 3A, TAG levels were higher in mouse LDAH-transfected cells than in control vector-transfected cells, and this observation was consistent whether non-tagged, GFP- or Flag-tagged LDAH were transfected. TAG levels also increased with transfection of human LDAH (Fig. 3B). Further supporting that LDAH promotes TAG accumulation, downregulation of endogenous LDAH using siRNA reduced TAG levels in OA-loaded cells (Fig. 3C). Interestingly, overexpressed LDAH considerably increased the amount of endogenous LDAH, which may indicate a self-positive autoregulation (Fig. 3B). OA treatment slightly increased LDAH levels (Fig. 3A–C). Thus, we asked how LDAH would respond under other situations of increased TAG deposition in LDs. Unlike the prototypical LD protein PLIN1, LDAH was readily detectable in undifferentiated 3T3-L1 cells, which is in line with the finding of ER-associated LDAH in the absence of lipid loading. However, LDAH levels significantly increased during 3T3-L1 differentiation into adipocytes (Fig. 3D). Next, we assessed a more physiologically relevant scenario of enhanced LD deposition by comparing LDAH expression between white adipose tissues of mice that were fed either regular chow or a Western-type diet for 4 weeks. As seen in Fig. 3E, LDAH levels were >3-fold higher in mice fed the high-fat diet, which may suggest a role in TAG accumulation *in vivo*. Thus, LDAH increases intracellular TAG levels, and the protein is upregulated under situations of enhanced TAG buildup in LDs.

**Figure 4:**
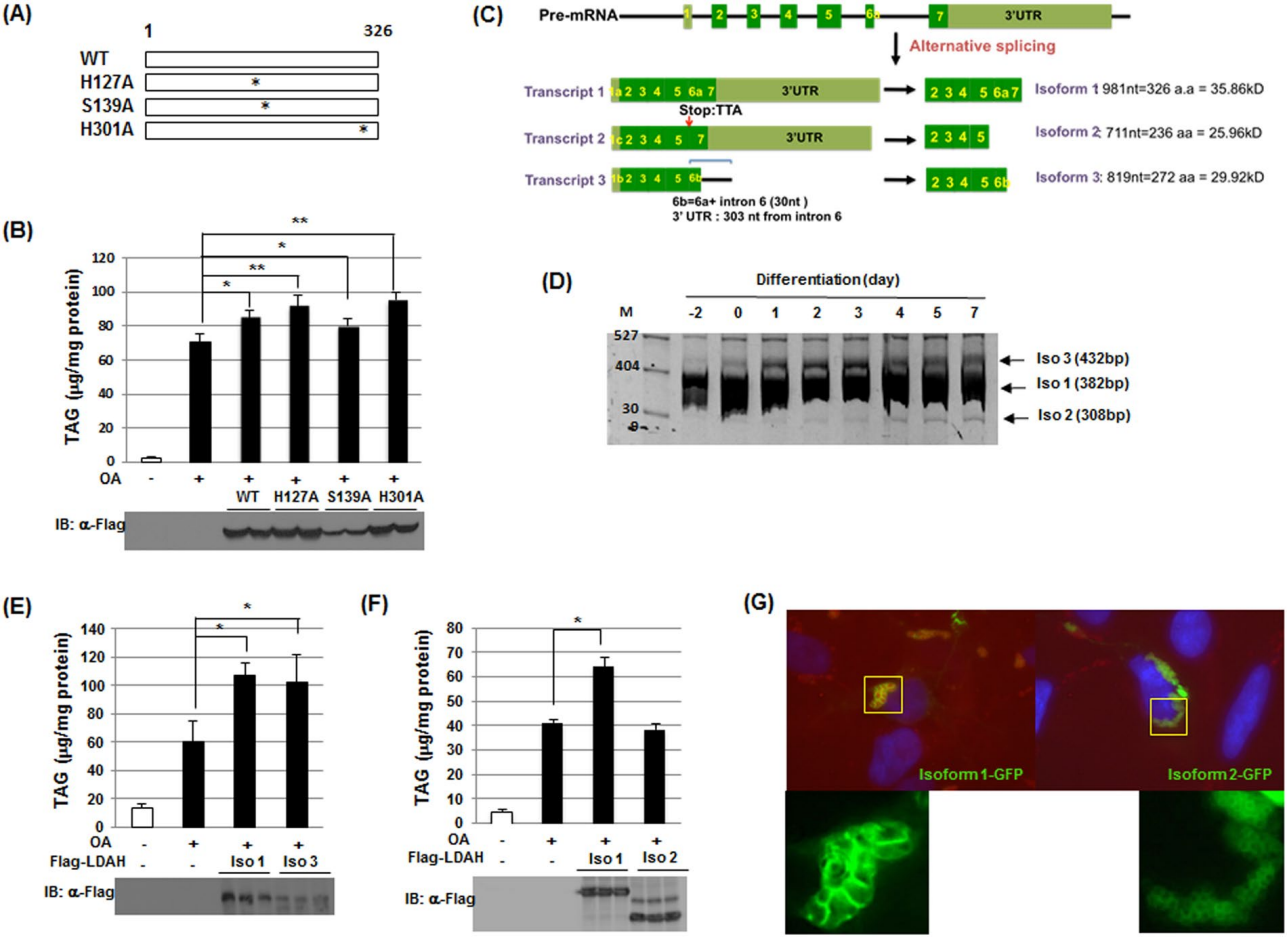
LDAH’s role in LD growth and TAG accumulation is related to its C-terminal region rather than to its catalytic sites. (**A**) Diagrams of point mutants of full-length LDAH used in the studies, including mutations of hystidine to alanine in predicted acyltransferase motifs, and of serine to alanine in the predicted hydrolytic motif. (**B**) TAG levels (top) and immunoblotting with anti-Flag antibody (bottom) in HEK293 cells overexpressing Flag-wild type or Flag-mutant LDAH, and loaded with OA (360 μM) for 24 h. *p < 0.05, **p < 0.005. (**C**) Diagram of predicted LDAH splicing variants. (**D**) mRNA expression of LDAH isoforms 1, 2, and 3 during 3T3L1 differentiation. (**E**) TAG levels in Flag-tagged LDAH isoform 1 and isoform 3 overexpressing cells stimulated with OA (360 μM) for 24 h. *p < 0.05. (**F**) TAG levels in Flag-tagged isoform 1 and isoform 2 overexpressing cells treated with OA (360 μM) for 24 h. *p < 0.05. Immunoblottings (bottom) were performed using a anti-Flag antibody. (**G**) LDAH isoform 1-GFP and LDAH isoform 2 -GFP were transfected to HEK293 cells. After 24 h cells were treated with OA (360 μM) for 24 h. LDAH was visualized by fluorescence microscopy (green) and LDs were stained with LipidTox (red).

The increase in TAG by LDAH is diametrally opposite of what would have been expected of a lipase. Thus, we performed an extended motif search analysis that revealed that in addition to the putative hydrolytic GxSxG motif, LDAH’s sequence harbors two motifs that might function in acyl transfer catalysis. Mouse LDAH’s carboxyl terminus includes a highly conserved HxxxxD sequence for acyltransferase activity in mammals (Figure S4)^22^. In addition, both mouse and human LDAH contain a central HxxxD sequence, a motif that has been shown to mediate acyltransferase activity in plants (Figure S4)^23^. To assess their role in TAG homeostasis, we generated point mutants to inactivate these two potential catalytic sites by exchanging histidine to alanine (H127A and H301A, Fig. 4A)^24^. However, TAG levels in cells transfected with either mutant increased to values similar to those of cells transfected with wild-type LDAH (Fig. 4B). Whether these motifs display low acyltransferase activity that induces modest changes on TAG that are not detectable under our experimental conditions, or might be involved in the generation of other lipid esters that have yet to be identified, remains unknown. However, our data suggest that they are not the primary drivers of the observed increase in TAG levels. We next speculated that the hydrolytic motif could indirectly impact TAG synthesis, for example by hydrolyzing other lipid esters to generate free fatty acids that could be recycled into TAG. Thus, we measured TAG levels after transfection of a mutant with the serine nucleophile replaced by alanine (S139A). Again, TAG levels were similar to the seen upon transfection of wild-type LDAH (Fig. 4B). In contrast, mutation of the active site serine to either alanine or cysteine did not decrease CE levels in cholesterol-laden HEK293 cells (Figure S5). Thus, the changes in TAG stores were not prevented by mutation of any of the potential active sites we identified.

In addition to full length LDAH (326 aa, 35.86 kDa), at least two other transcripts are predicted as a result of combinational alternative splicing at the 5′ UTR and C-terminus of LDAH’s pre-mRNA (Fig. 4C and Figure S4B). Isoform 2 is the shortest splicing variant, and is missing 90 C-terminal amino acids (236 aa, 25.96 kDa). Isoform 3 is shorter (272 aa, 29.92 kDa), and contains a different C-terminus than isoform 1 (Fig. 4C and Figure S4B). PCR analysis using primers to identify each isoform (Figure S6) during differentiation of 3T3-L1 cells into adipocytes showed that transcripts coding for isoform 1 are most abundant and readily detectable even in preadipocytes (Fig. 4D). Transcripts coding for isoforms 2 and 3 were very low in preadipocytes. Their levels increase during differentiation, but remain much lower than those of isoform 1 (Fig. 4D). To assess the impact of each isoform on TAG accumulation, we transfected the Flag-tagged isoforms and stimulated HEK293 cells with OA (360 μM for 24 h). While LDAH isoform 3 increased TAG to a similar extent than isoform 1 (Fig. 4E), isoform 2 did not affect TAG content (Fig. 4F). Interestingly, isoform 2 still localized to LDs, including clustered LDs, but it did not promote LD fusion (Fig. 4G). Taken together, these data indicate that LDAH’s C-terminus is not necessary for its association with LDs, but this region is critical for LDAH to mediate LD fusion and increase TAG accumulation.

### LDAH enhances TAG biogenesis and inhibits TAG turnover

**Figure 5:**
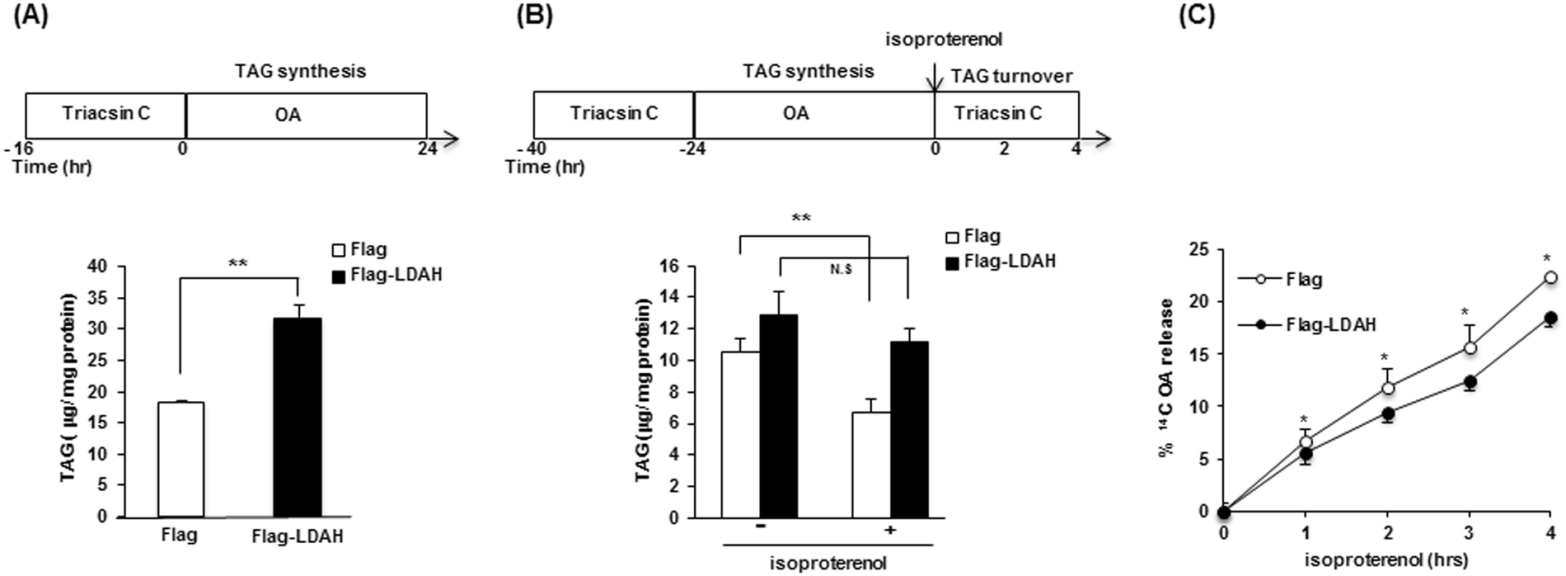
LDAH increases TAG synthesis and decreases TAG hydrolysis. HEK293 cells were transfected with Flag or with Flag-LDAH. Cells were synchronized with triacsin C (1 μg/ml) for 16 h in delipidated media, followed by treatment with OA for 24 h in the absence of triacsin C. (**A**) Intracellular TAG levels were determined and normalized to protein. **p < 0.005. (**B**) TAG hydrolysis was induced by incubation with isoproterenol (10 μM) for 2 h in the presence of triacsin C. TAG content was measured and normalized to protein. Two-way ANOVA showed that both LDAH and isoproterenol affect TAG levels. However, the effect of isoproterenol depends on whether LDAH is present, being significantly reduced at p = 0.002 in control-transfected cells but not significantly reduced (p = 0.064) in cells overexpressing LDAH. Relevant pairwise comparisons are showed in the Figure. **p < 0.005; N.S. = not significant. (**C**) Cells were loaded with ^14^C-labeled OA following the protocol outlined in panel B, the amount of OA released to the culture media following isoproterenol (1 μM) treatment was determined by scintillation counting. The data represents the % of OA released relative to the total amount of OA (intracellular + extracellular). *p < 0.05.

The increase in TAG content by LDAH could owe to changes in biogenesis, in hydrolysis, or in both processes. To investigate LDAH’s role during conditions of lipid biogenesis, we performed a pulse-chase experiment in which cells were first cultured in delipidated media and synchronized for 16 h with triacsin C (1 μg/ml), a drug that blocks TAG synthesis^25, 26^. Cells were then treated with OA (360 μM for 24 h) in the absence of triacsin C, and intracellular TAG was quantified^26^. As seen in Fig. 5A, TAG levels in cells overexpressing LDAH were higher than in control-vector transfected cells, suggesting increased biogenesis. To assess LDAH’s effect during TAG hydrolysis, cells were cultured in delipidated media containing triacsin C for 16 h, and incubated with OA (360 μM) for an additional 24 h in the absence of triacsin C. After lipid loading, TAG hydrolysis was induced by treatment with isoproterenol (10 μM), a β-adrenergic agonist, in the presence of triacsin C to inhibit recycling of the released free fatty acids into TAG. Interestingly, 2 h of β-adrenergic stimulation significantly reduced TAG levels in control-transfected cells, whereas in cells transfected with Flag-LDAH the response to isoproterenol was modest and did not reach statistical significance (Fig. 5B). To assess whether these changes in TAG turnover would be reflected in changes in free fatty acid release from cells, we loaded cells with ^14^C-labeled oleate following the protocol outlined in Fig. 5B, and determined the amount of ^14^C-oleate released to the media. In line with the data from TAG turnover experiments, we observed a lower rate of oleic acid release to the culture media in LDAH overexpressing cells (Fig. 5C). In general, these data suggest not only that LDAH does not mediate TAG hydrolysis, but also that it actually ameliorates the process.

### LDAH promotes ATGL degradation by the proteasome

**Figure 6:**
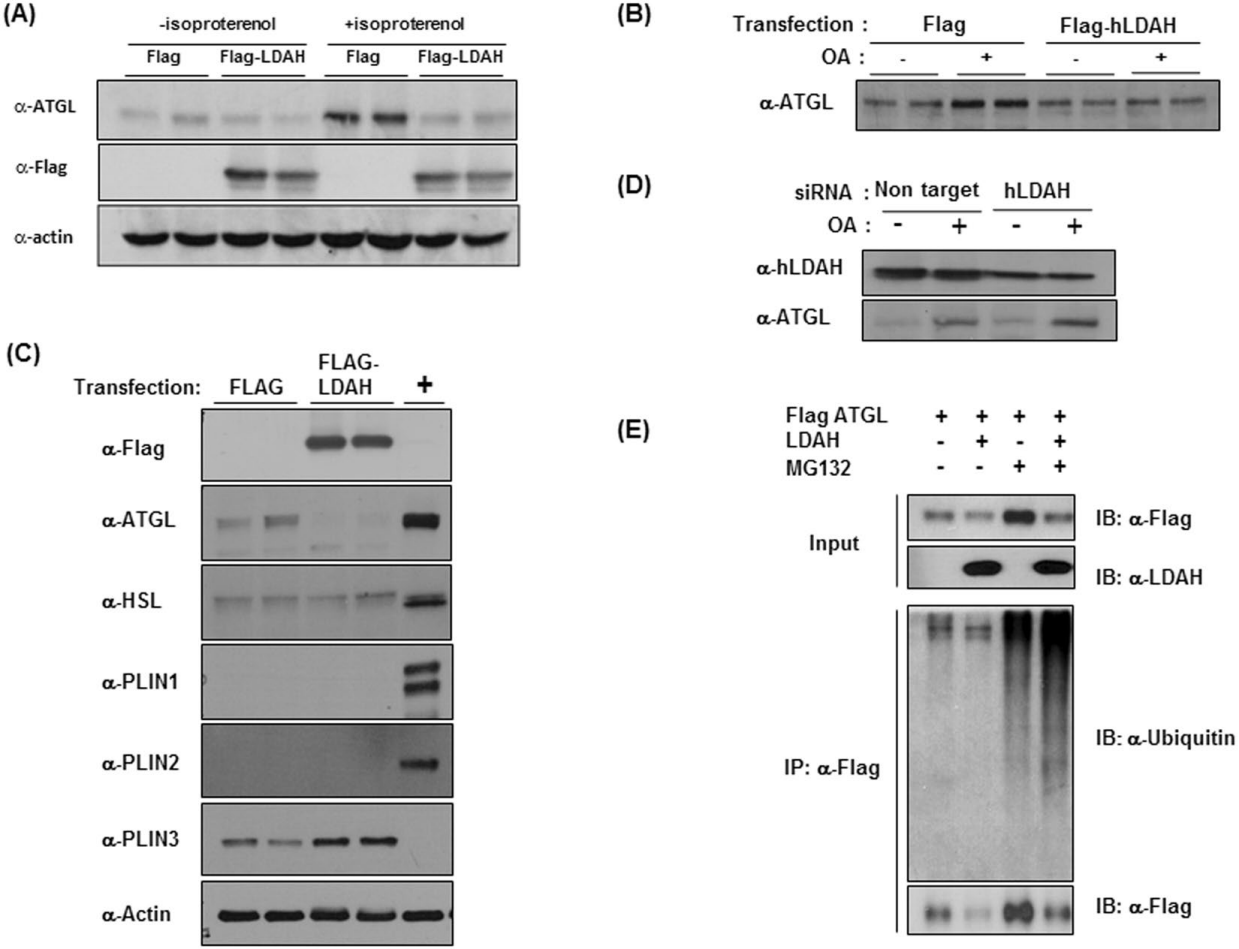
LDAH decreases the levels of ATGL. (**A**) ATGL levels in Flag or Flag-LDAH transfected HEK293 during TAG hydrolysis as described in Fig. 5B. (**B**) Flag or Flag-human LDAH (hLDAH) were transfected to HEK293 cells, and cells were cultured with or without OA (360 μM) for 24 h. ATGL was detected using αnti-ATGL antibody. (**C**) Flag or Flag-LDAH were transfected to HEK293 cells. Cells were stimulated with OA for 24 h, and HSL, PLIN1, PLIN2, PLIN3 and ATGL were detected by immunoblotting with specific antibodies. Anti-actin antibody was used for loading control. Differentiated 3T3L1 adipocytes (+) were used for expression control. (**D**) Non-target or hLDAH siRNA were transfected to HEK293 cells. After 48 h OA (360 μM) was added to the culture media for 24 h. The expression levels of hLDAH and ATGL were determined by immunoblotting. (**E**) Flag-ATGL and/or LDAH were transfected to HEK293 cells as indicated. 24 h after transfection cells were treated with OA (360 μM for 24 h) followed by MG132 for 8 h as indicated in the figure. Flag-ATGL was pulled-down with an anti-Flag M2, and immunoblots were performed with anti-ubiquitin, anti-Flag, and anti-LDAH antibodies.

ATGL catalyzes the initial rate-limiting step in TAG hydrolysis^27, 28^. Thus, we asked whether the changes in intracellular TAG content, particularly the lower rates of hydrolysis, could be related to changes in ATGL levels. Strikingly, we found that ATGL levels increased considerably in control-transfected cells treated with isoproterenol, but not in LDAH overexpressing cells (Fig. 6A). Isoproterenol promotes ATGL redistribution to the LD surface, and several studies reported that ATGL bound to LDs is stabilized because it is protected from the ubiquitin-proteasome degradation pathway^29–32^. Consistent with these reports, we also observed higher ATGL levels in OA-treated cells (Fig. 6B)^26^. However, in line with the observations in isoproterenol-treated cells, ATGL levels did not increase in cells transfected with Flag-LDAH (Fig. 6B). We considered the possibility that the decrease in ATGL could be related to a general effect of LDAH overexpression on other LDAPs. One of the best-characterized families of LDAPs is the perilipin (PLIN) family^33, 34^. As previously reported, we did not detect PLIN1 and PLIN2 in HEK293 cells, while PLIN3 was readily detectable (Fig. 6C)^35^. However, in contrast to ATGL, PLIN3 levels were actually higher in LDAH overexpressing cells. In addition, levels of endogenous hormone-sensitive lipase (HSL), another LD exchangeable lipase, were not affected by LDAH overexpression, suggesting a distinct effect of LDAH on the regulation of ATGL levels (Fig. 6C). Further supporting that LDAH is a negative regulator of ATGL, ATGL levels increased upon siRNA-mediated downregulation of endogenous LDAH (Fig. 6D).

ATGL is degraded by the ubiquitin-proteasome pathway^26, 36^. To assess whether LDAH impacts this process, we transfected HEK293 cells with Flag-ATGL, with untagged LDAH, or with the combination of both plasmids. Cells were then loaded with OA (360 μM for 24 h), and remained untreated or were treated with MG132 for 8 h to inhibit the activity of the 26 S proteasome complex. Flag-ATGL was then pulled-down, and ubiquitin, Flag-ATGL, and LDAH were detected by immunoblotting. As anticipated, treatment with MG132 resulted in accumulation of polyubiquitinated ATGL. However, polyubiquitinated products were more abundant in cells co-transfected with LDAH, suggesting that LDAH enhances ATGL ubiquitination and proteasomal degradation (Fig. 6E). Consistently, the decrease in ATGL seen under LDAH overexpression was reduced by treatment MG132 (Fig. 6E). In agreement with the observation that LDAH isoform 2 did not increase TAG stores, this isoform did not affect ATGL ubiquitination and did not reduce ATGL protein levels (Figure S7). Overall, these results indicate that LDAH may indirectly regulate intracellular TAG stores by decreasing the stability of ATGL, and that this regulatory mechanism is not shared by LDAH isoform 2.

### Functional reciprocity between LDAH and ATGL

**Figure 7:**
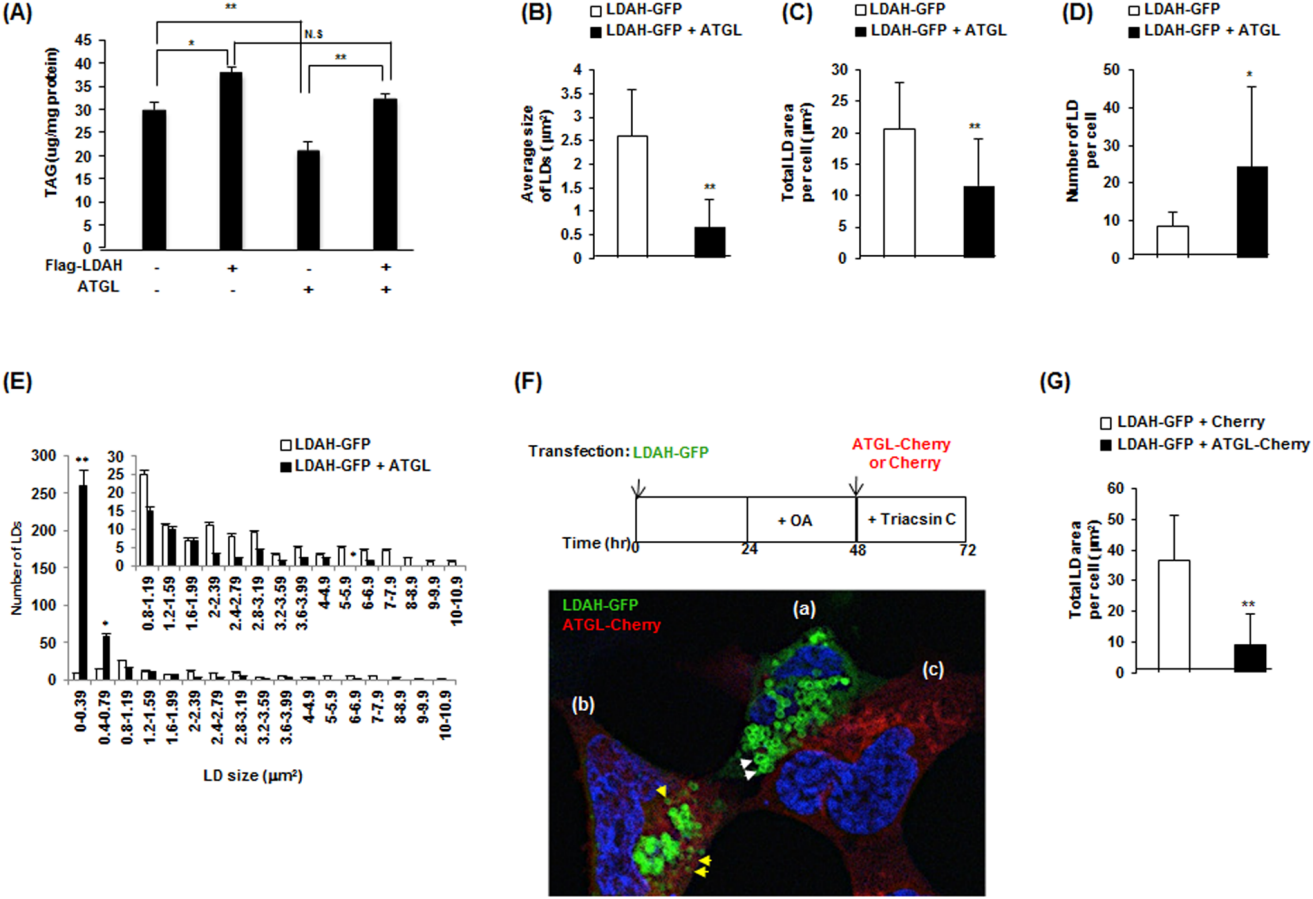
LDAH and ATGL display antagonistic effects. (**A**) Flag-LDAH and/or ATGL were transfected to HEK293 cells as indicated. TAG was measured after 24 h of OA (360 μM) loading and normalized to protein. ANOVA analysis confirmed a significant effect of both LDAH and ATGL on intracellular TAG stores at p < 0.001. Pairwise comparisons are shown in the chart. *p < 0.05, **p < 0.005; N.S. = not significant. **(B–E)** LDAH-GFP was co-transfected with Flag or Flag-ATGL to HEK293 cells, and cells were treated with OA (360 μM) for 24 h. (**B**) Average size of LDs per cell; (**C**) total area of LDs per cell; (**D**) number of LDs per cell; and (**E**) size distribution of LDs. Fifteen to sixteen cells per group were used for the measurements. *p < 0.05, **p < 0.005. (**F**) Diagram of experimental approach (top). LDAH-GFP transfected cells were treated with OA (360 μM) for 24 h. Cells were then transfected with ATGL-cherry in the presence of triacsin C (1 μg/ml). Expression of LDAH-GFP alone (a) and ATGL-cherry alone (c), and co-expression of LDAH-GFP and ATGL-cherry (c) were visualized by confocal microscopy. White arrows: LDs in cells expressing only LDAH-GFP. Yellow arrows: LDs in cells expressing both LDAH-GFP and ATGL-cherry. (**G**) Quantification of LD area per cell in cells overexpressing both LDAH-GFP and Cherry, or both LDAH-GFP and ATGL-Cherry (n = 14 per group). **p < 0.005.

To further examine the interrelationship between LDAH and ATGL, we transfected HEK293 cells with Flag-LDAH, ATGL, or the combination of both plasmids, and treated the cells with OA (360 μM, 24 h). As anticipated, cells transfected with LDAH or ATGL displayed higher and lower TAG content, respectively. However, supporting an antagonistic relationship between the two proteins, co-transfection of both LDAH and ATGL resulted in a nearly neutral effect on TAG levels (Fig. 7A). Arguably the most distinctive effect of LDAH is the induction of changes in LD architecture. Interestingly, ATGL reverted these changes. The average LD size and total LD area were significantly lower in cells co-transfected with ATGL and Flag-LDAH than in cells transfected with Flag-LDAH alone (Fig. 7B and C). Co-transfection of ATGL also resulted in a very robust increase in the number of cytosolic LDs, particularly of small droplets (Fig. 7D and E). Overall, these results suggest an antagonistic relationship between LDAH and ATGL both on TAG stores and LD phenotype. Given that LDAH readily coats newly formed LDs, we next asked whether under a scenario in which LDs are already coated with LDAH, ATGL would still be able to access and mobilize lipid from preexisting LDAH associated LDs. To answer this question, first we transfected HEK293 cells with LDAH-GFP and treated them with OA for 24 h to allow for LDAH enrichment at the LD surface. Then cells were transfected with either cherry or ATGL-cherry in the presence of triacsin C to inhibit generation of new LDs. Of note, in general LDs were smaller in triacsin C-treated cells than cells in regular culture media. Approximately 40% of cells co-expressed LDAH and ATGL. As seen in Fig. 7F, ATGL-cherry signal was also detected at the LD when LDAH-GFP was associated with LDs (Fig. 7F-b). LD size in cells co-expressing ATGL and LDAH (Fig. 7F-b) was smaller than in cells expressing only LDAH (Fig. 7F-a). In addition, morphometric analyses showed that ATGL was able to significantly reduce the area of LDs in cells whose LDs were pre-coated with LDAH (Fig. 7G). Altogether, the data indicate that LDAH and ATGL counterbalance their effects on TAG stores, and that ATGL is able to reverse the changes in LD phenotype induced by LDAH, and to access and act on LDs pre-coated with LDAH.

## Discussion

The structure and function of LDs are governed by LDAPs that transiently or constitutively associated with them^9, 37^. LDAH has recently been identified in LD fractions, and is highly expressed in TAG-rich tissues such as white and brown adipose tissues and liver^14–17^. Here we also observed higher LDAH expression in differentiated 3T3-L1 adipocytes than in preadipocytes and, importantly, a measurable increase in LDAH expression in expanding white adipose tissue of mice fed a Western diet. Although LDAH’s sequence contains a prototypical esterase/lipase motif, it did not display TAG hydrolase activity, suggesting that its main role is not in promoting lipolysis^14, 15^. Indeed, our overall data suggest that under conditions of TAG deposition in LDs LDAH plays a role opposite to what would be expected from a lipase, as it facilitates LD growth and TAG accumulation. We found that LDAH promotes fusion and enlargement of clustered LDs, resulting in changes in LD architecture that are very similar to the previously described in the Drosophila ortholog of LDAH (CG9186) by Thiel *et al*.^14^. An immediate question after this finding was whether LDAH increased TAG storage. Supporting this notion, we have consistently observed increased TAG levels in LDAH isoform 1 transfected cells. However, there were no changes in TAG levels in cells transfected with LDAH isoform 2, which was still associated to clustered LDs but did not induce LD fusion.

Depending on metabolic conditions, cytosolic LDs undergo dynamic changes in size and number. LD size is in turn one of the parameters that regulate the rates of lipid storage and mobilization. While smaller LDs provide higher surface area for lipases, larger LDs provide a lower surface to volume ratio, and are therefore considered a more efficient form of fat storage^11^. LD fusion is one of the mechanisms that have been proposed to mediate LD expansion. While still not well understood, fusion between LDs is not only regulated by lipids but also by LDAPs^11^. For example, among LDAPs, Fat specific protein 27 (Fsp27/CIDEA) is enriched at LD-LD contact sites, and mediates directional lipid transfer from small to large LDs^11, 13, 38^. The mechanisms that regulate the fusion of larger LDs of similar size, known as homotypic fusion, still remain unclear^11^. A recent report identified rapid and microtubule-dependent homotypic fusion in reformation of large LDs in adipocytes treated with insulin and OA^21^. Several SNARE proteins were shown to promote LD fusion, including fusion of droplets of similar size in a process that was also dependent on intact microtubules^20, 38^. The coalescence of similarly sized LDs induced by LDAH is also highly suggestive of a role of the protein in homotypic fusion.

Enhanced LD growth could by itself increase TAG stores. However, an intriguing observation was that LDAH not only increased TAG during LD biogenesis, but also decreased TAG turnover and oleic acid release from cells. This raised the possibility of an indirect effect that could involve interference with the action of lipases. ATGL, the first rate-limiting enzyme in TAG lipolysis, is a short-lived protein, and the rates of lipolysis are proportional to the levels of ATGL protein^39^. Indeed, ATGL is up-regulated by fasting, when active lipolysis is needed, and down-regulated by insulin, a hormone that promotes TAG storage^40, 41^. In addition, some proteins that increase lipid storage, such as Fsp27/CIDEA, have been shown to inhibit lipolysis by negatively regulating ATGL^42, 43^. Thus, we presumed that this could be the mechanism by which LDAH ameliorates TAG turnover. This possibility was supported by the findings that LDAH decreased ATGL levels. The reduction in ATGL protein was dramatic under treatment with a β-adrenergic agonist, which increases ATGL localization to LDs to mediate lipolysis^44^. ATGL is degraded by the ubiquitin-proteasome system, and this regulatory mechanism has been shown to influence obesity, as well as hepatic ATGL content and TAG turnover in fatty liver^36, 45^. Higher levels of polyubiqutinated ATGL under LDAH overexpression identified increased proteasomal degradation as a likely mechanism by which LDAH decreases ATGL levels. How LDAH promotes ATGL degradation is not clear. However, it is known that when bound to LDs ATGL is protected from degradation^26, 30^. It was previously shown that protein crowding is a determinant of LD protein composition. Interestingly, CG9186 displayed higher affinity for LDs than Brummer, ATGL’s drosophila ortholog^14, 46^. Thus, a possible explanation for the reduction in ATGL could involve competition for LD binding sites, resulting in increased cytoplasmic localization and degradation of ATGL. Nevertheless, the facts that co-transfection of ATGL antagonizes the changes in TAG stores and LD morphology induced by LDAH, and that ATGL is able to reduce the size of LDs pre-coated with LDAH, suggest that LDAH does not totally outcompete ATGL at the LD surface. The finding that one of LDAH’s isoforms that also localizes at the LD surface does not affect ATGL ubiquitination, ATGL protein levels, and TAG levels, also suggests a complex interrelationship between LDAH and ATGL beyond their affinity for LDs.

The relevance of LDAH in lipid metabolism *in vivo* has been evaluated. Thiel *et al*. reported that knockdown of CG9186 using siRNA decreased stored TAG in 6 days old fly. This suggests a lipogenic involvement of CG9186 in TAG metabolism *in vivo*, although they found that overexpression of CG9186 in *Drosophila* Kc167 cell line had no effect on TAG levels but it generated clustered and fused LDs like mammalian LDAH^14^. Kory *et al*. recently published that systemic deletion of LDAH in mice did not induce significant differences in multiple lipid parameters in liver and adipose tissues^47^. As authors discussed, it is possible that LDAH is either not involved in lipid metabolism *in vivo* or that redundancy might mask the loss of LDAH, which is sometimes reported in case of LDAPs^9, 48^. The ability of organisms to store energy is critical to sustain life in times of nutrient deprivation. Hence, multiple enzymes with often seemingly redundant functions tightly regulate the processes of lipid storage and mobilization. Such complexity has challenged the characterization of some of the players, many of which are proteins that associate with LDs. Further research will be needed to determine whether functional redundancy might compensate for the loss of LDAH *in vivo*, or LDAH modifies LD metabolism by acting on different substrates that have yet to be identified. The data presented in this manuscript suggests that future studies to elucidate LDAH’s function should focus on metabolic conditions that promote TAG storage in LDs. An important remark is that while this study focus primarily on measurements of TAG stores and free fatty acid release, there are other important processes that might also be affected by changes in lipid flux in response to LDAH, including changes in fatty acid signaling or on the channeling of free fatty acids to the mitochondria for β-oxidation^49–51^.

In conclusion, this report supports that under OA loading to promote TAG storage in LDs LDAH plays a primarily lipogenic role. The induction of LD growth and TAG accumulation are independent of potential catalytic and acyltransferase motifs, but rely on the protein’s C-terminal sequence. The results also suggest that the increase in TAG levels could, at least in part, be attributed to a reduction in ATGL stability.

## Materials and Methods

### Reagents and antibodies

Custom polyclonal rabbit anti-human and mouse LDAH antibodies were generated at Bethyl Laboratories, and characterized as previously described^15^. Bodipy 493/503, LipidTox, and RNAiMAX were from Invitrogen. Fugene HD was purchased from Promega. Isoproterenol hydrochloride, anti-beta actin antibody, anti Flag-M2-HRP, anti Flag-M2-agarose, mouse IgG, oleic acid, 3-isobutyl-1-methylxanthine, insulin, dexamethasone and nocodazole were from Sigma-Aldrich. Triacsin C and anti-ubiquitin antibody were from Santa Cruz Biotechnology. Anti-PLIN1 antibody was from Progen. Anti-beta tubulin and anti-PLIN2 antibodies were from Nobus Biologicals. Anti-PLIN3 was purchased from Fitzgerald. Anti-ATGL and anti-HSL were from Cell Signaling. Alexa Fluor 647-conjugated secondary antibodies and anti-GM130 were from BD Pharmingen. Protein G-agarose and protein-A-agarose were from Thermo Scientific. MG132 was purchased from Calbiochem. Total and free cholesterol were measured using kits from Wako. TAG were determined with Infinity Trglyceride reagents from Thermo Scientific. ^14^C-oleic acid was purchased from Perkin Elmer.

### DNA plasmids and siRNAs

Mouse Flag-LDAH constructs were generated as we previously described^15^. Mouse LDAH isoform 2 (pYX-ASC LDAH2) was purchased from Thermo Scientific, and the PCR amplified isoform was subcloned into pCMV10 3xflag vector using NotI/XbaI sites. Human LDAH (pOBT7 C2orf43) from GeneCopoeia was amplified by PCR and subcloned into pCMV10 3xflag vector. To generate LDAH isoform 2-GFP, the PCR amplified isoform was subcloned into pEGFPN1 vector using XhoI/BamHI sites. To generate LDAH isoform 3, cDNA libraries were prepared from Raw 264.7 macrophages and C57BL6/T mouse liver RNAs using Superscript II reverse transcriptase (Invitrogen). To amplify LDAH isoform 3, PCR was performed using the forward primer (5′-GAACTTCCGGGTCTGGTGAG-3′) and the reverse primer (5′-CCATTCTTGCCTTGTGTCCT-3′) using the cDNA libraries as template. PCR products were purified and sequenced. PCR-confirmed isoform 3 obtained from cDNAs of mouse liver and Raw 264.7 macrophages completely matched with the transcript variant 3 (which codes for LDAH isoform 3) listed in Genbank (NM_001167767.1). This PCR product was used as a template to subclone LDAH isoform 3 to pCMV10 3xflag vector using XbaI/NotI sites. LDAH point mutations in full-length LDAH were generated by 2 step PCR, and mutants were subcloned to pCMV10 3xflag vector using NotI/XbaI sites. Mouse ATGL (pCMNK-ATGL) was kindly provided by Dr. Weiqin Chen, at Georgia Regents University. To generate flag-tagged ATGL, ATGL was amplified by PCR and subcloned into pCMV10 3xflag vector using HindIII/NotI sites. To generate ATGL-cherry, PCR-amplified ATGL was subcloned to pmCherry N1 vector (Clontech) using HindIII/AgeI sites. pEGFPN1-mouse LDAH and pCMV-SPORT mouse LDAH were described previously^15^. pDsRed2-ER was provided by Dr. Yunfei Huang at Albany Medical College. pCMV SPORT6 mouse HSL was purchased from Thermo Scientific. Flag-PLIN2 was generated by subcloning PCR amplified mouse PLIN2 to pCMV10 3xflag vector using HindIII/XbaI sites. Human LDAH siRNA and non-target siRNA were from Ambion (sense 5′-GGACAUUUAUGGACUAAAUtt-3′ antisense 5′-AUUUAGUCCAUAAAUGUCCtt-3).

### Cell culture and transfection

HeLa and HEK293 cells were purchased from ATCC and cultured in DMEM (Invitrogen) supplemented with 10% fetal bovine serum (Sigma-Aldrich) and 1% antibiotic-antimycotic (Invitrogen) unless otherwise stated. In some cases the cell culture medium was supplemented with OA complexed to bovine serum albumin at a 7.7:1 ratio. Additional reagents were used as indicated in figure legends. For plasmid transfection, cells were plated and maintained in culture for a day. Media were refreshed prior to transfection. Transfection was performed using Fugene HD (Promega) according to the manufacturer’s instructions. For siRNA transfection, 1 × 10^5^ HEK293 cells were seeded in 6-well plates and transfected with 20 nM of either non-target or human LDAH siRNA using RNAiMax (Invitrogen). 3T3L1 preadipocytes were differentiation into adipocytes by differentiation media (1 μM dexamethasone, 0.5 mM 3-isobutyl-1-methylxanthine, and 10 μg/ml insulin). From the 2^nd^ day of differentiation, cells were cultured in regular culture media supplemented with insulin (10 μg/ml). From the 4^th^ day of differentiation, cells were cultured in regular culture media until day 7.

### Cellular lipid measurements

Approximately 1.3 to 1.5 × 10^5^ HEK293 cells were cultured for a day in 6-well culture dishes. After transfection, cells were maintained for 24 h (for plasmids) or 48 h (for siRNAs). Culture media were refreshed, and cells remained untreated or were treated with OA for 24 h at the doses indicated in the figure legends. Cells were rinsed and harvested with 1xPBS (pH 7.4). Cellular lipids were extracted with hexane:isopropanol (3:2, vol/vol), air-dried under nitrogen gas, and re-dissolved in 2% Triton X-100 in isopropanol. TAG were biochemically measured with Infinity Triglyceride reagents. Following lipid extraction, cellular protein pellets were dissolved in 0.1 N NaOH and briefly sonicated. Protein concentrations were determined with DC-protein assay (Bio-Rad) and used to normalize TAG levels. For cholesterol measurements, HEK293 cells were treated and processed as we previously described^15^.

### TAG biogenesis and hydrolysis assays

To determine the effect of LDAH during conditions of TAG biogenesis, cells were synchronized by replacing the culture media by DMEM supplemented with 10% charcoal-striped FBS and triacsin C (1 μg/ml) for 16 h. Cells were rinsed with sterile PBS, and oleic acid was added to the culture media to a final concentration of 360 µM to promote TAG biogenesis. Intracellular TAG levels were determined after 24 h and normalized to protein. To study the role of LDAH under conditions of hydrolysis, following synchronization with triacsin C and lipid loading with OA, hydrolysis was initiated by treatment with isoproterenol (10 µM). After 2 h, lipid and protein were extracted and TAG were measured as described above. For the free fatty acids efflux assay, the TAG hydrolysis protocol was applied as described above with the only exception that HEK293 cells cultured in 12- well plates were loaded with 2 μM of ^14^C-oleic acid mixed with 360 μM of cold-oleic. Aliquots of media were collected every hour and centrifuged to remove cell debris. Radioactivity in the aliquots of media and total cells, lysed in 0.1 N NaOH, were measured in a liquid scintillation counter, and the percent of ^14^C-oleic acid released to the media was calculated.

### Immunofluorescence

Cells were cultured on coverslips or in 6-channel μ-slides (Ibidi) for one day. Plasmids were transfected using Fugene HD (Roche) as described in Figure Legends. After 24 h, cells remained untreated or were treated as indicated in the manuscript text and Figure Legends. Cells were fixed with 3.7% paraformaldehyde in 1xPBS (pH 7.4) and permeabilized with 0.1% saponin or with 0.3% Triton X-100 in 1xPBS. GFP-tagged constructs (green) were directly detected by fluorescence microscopy, using LipidTox (red) and DAPI (4′,6-diamidino-2-phenylindole, blue) to visualize LDs and cell nuclei, respectively. Flag-tagged constructs were detected by immunocytochemistry with anti-flag antibody, followed by Alexa Fluor 647-conjugated secondary antibody. Images were taken with a Zeiss Observer.D1 microscope or with a Zeiss LSM 510 META confocal microscope.

### Immunoprecipitation and immunoblotting

Cells cultured in 10 cm culture dishes were transfected and/or treated as indicated in Figure Legends. To obtain protein lysates, cells were rinsed with 1xPBS (pH 7.4) and lysed in RIPA buffer (25 mM Tris-HCl, pH 7.5, 150 mM NaCl, 1% Nonidet P-40, 10% glycerol, and 10 mM EDTA) with protease inhibitor cocktail (Roche). Cells were centrifuged at 20,000 × g at 4 °C for 15 min, and supernatants were collected and used for immunoblotting. For co-immunoprecipitation experiments, 24 h after transfection with Flag-ATGL, with pCMV-SPORT-LDAH, or the combination of both plasmids, cells were treated with OA (360 μM) for 24 h. Media were replaced by DMEM-10% FBS and cells were treated with MG132 (10 μM) for 8 h as indicated in Fig. 6. Cells were lysed in RIPA buffer, and 20,000 × g supernatants were pre-cleared with 30 µl of protein G-agarose under rotation at 4 °C for 3 h. Pre-cleared lysates (200 μg of protein) were immunoprecipitated with either anti-flag M2-agarose or control mouse IgG overnight under rotation at 4 °C. The anti-flag M2-agarose was pelleted by centrifugation at 20,000 × g at 4 °C for 5 min, and washed 5 times with RIPA buffer. Immunoprecipitated proteins were dissociated from the M2-agarose with SDS-PAGE loading buffer. Immunoblottings were performed as indicated in Fig. 6, using 5% of total lysates for input. For immunoprecipitaion of isoform 2, pCMX-ATGL and Flag-LDAH-isoform 2 were transfected as described in the figure. Immunoprecipitation was performed with anti-ATGL antibody and protein A-agarose. For LDAH western blot in adipose tissues, C57BL6 female mice from Taconic were fed regular mouse chow for 14 weeks, or mouse chow for 10 weeks and a western type diet containing 21% milk fat and 0.15% cholesterol (Teklad TD.88137) for an additional 4 weeks. At 14 weeks mice were fasted overnight, sacrificed, and white adipose tissues snap frozen in liquid nitrogen. Protein homogenates were prepared in RIPA buffer, and used for immunoblotting. All procedures on live animals were conducted following protocols approved by the Institutional Animal Care and Use Committee at Albany Medical College.

### Statistical analysis

Statistical analysis was performed using two-tailed Student’s t-test. Two-way ANOVA followed by pairwise comparisons with the Tukey’s HSD test was used for two-way comparisons. Differences were considered significant when p < 0.05. Unless otherwise stated, only statistically significant comparisons are indicated in the figures. All experiments were performed at least in triplicate. Data are expressed as mean ± SD.

## Electronic supplementary material

Supplementary material
