# Supplementary material for: Lipid Droplet-Associated Hydrolase Promotes Lipid Droplet Fusion and Enhances ATGL Degradation and Triglyceride Accumulation
Source: Sci Rep. 2017 Jun 2;7:2743. doi: 10.1038/s41598-017-02963-y (PMC5457427; doi:10.1038/s41598-017-02963-y)

Lipid Droplet-Associated Hydrolase Promotes Lipid Droplet Fusion and Enhances ATGL Degradation and Triglyceride Accumulation

Young-Hwa Goo[1,*], Se-Hee Son[1], and Antoni Paul, PhD[1,*]

From [1]Department of Molecular and Cellular Physiology, Albany Medical College, Albany, NY

*Correspondence to Young-Hwa Goo or Antoni Paul. Department of Molecular and Cellular Physiology, Albany Medical College, 47 New Scotland venue, MC-8, Albany, NY 12208, USA. E-mail: gooy@mail.amc.edu or paula@mail.amc.edu

SUPPLEMENTAY INFORMATION

1. Supplementary Figures:

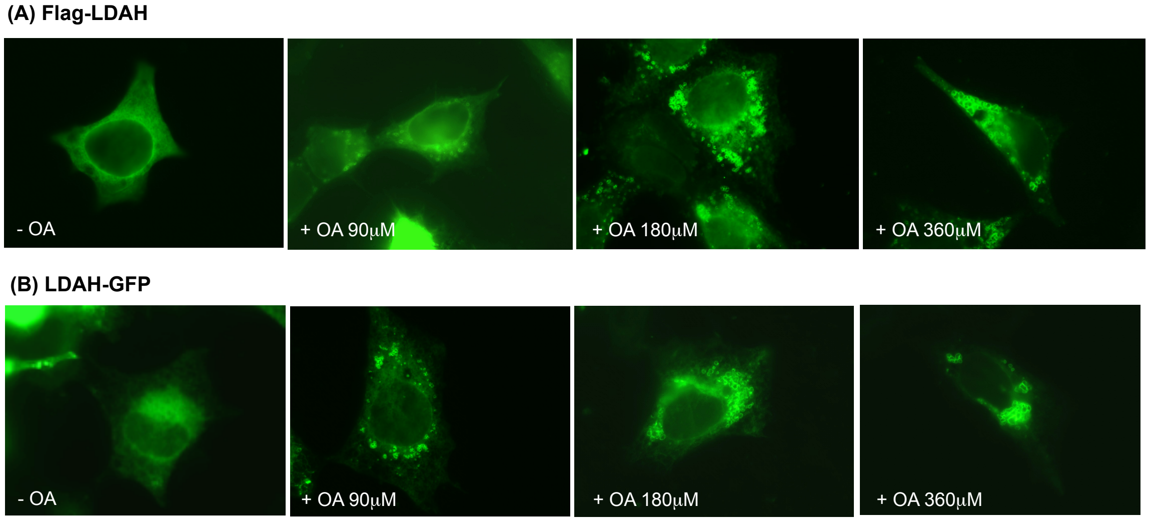

Supplementary Figure. 1. Both Flag- and GFP-tagged LDAH localize to LDs, and induce changes in LD morphology that are more pronounced at higher dose of OA. Flag- or GFP-tagged LDAH were overexpressed in HEK 293 cells. Cells were cultured for 24 h under different doses of OA (0, 90, 180, and 360 μM). Anti-Flag antibody (green) was used for immunofluorescence to detect Flag-LDAH.

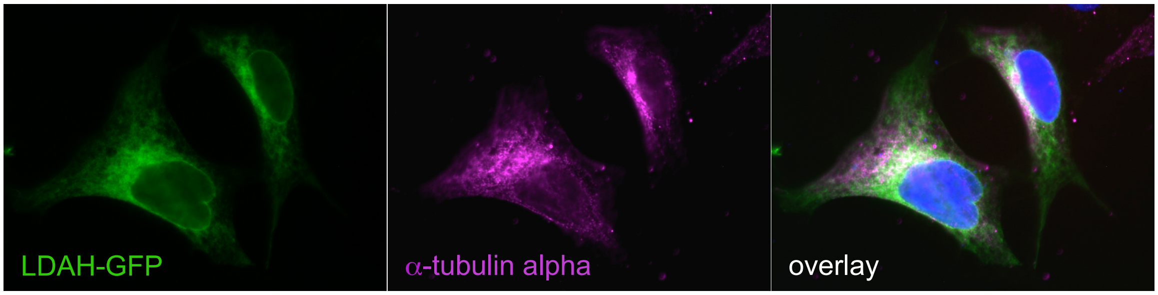

Supplementary Figure 2. Immunofluorescence showed some degree of colocalization between LDAH and microtubules. LDAH-GFP expressing HEK293 cells (green) were stained with anti-tubulin (magenta) antibodies.

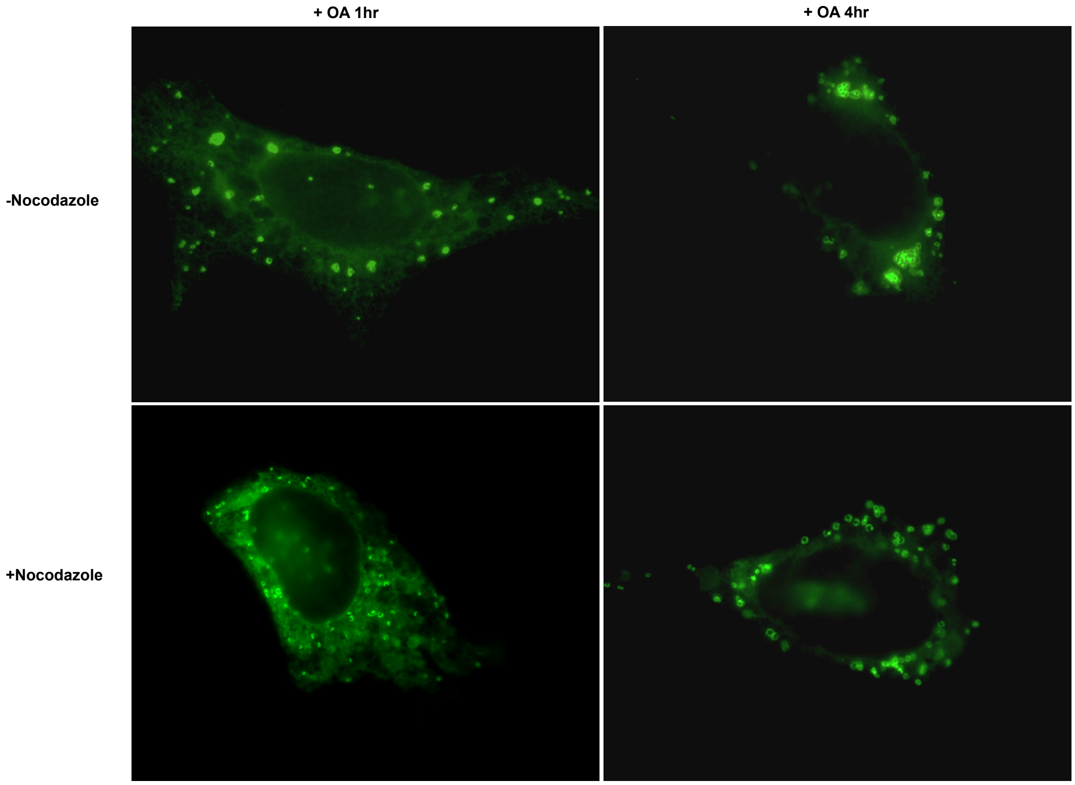

Supplementary Figure 3. Nocodazole does not block LDAH association with LDs. HEK293 cells expressing LDAH-GFP (green) remained untreated or were pretreated with nocodazole (2 μg/ml) for 30 min, followed by treatment with OA (360 μM) in the presence or in the absence of nocodazole for 1 or 4 h.

(A)

```
D. melanogaster          1   ----------MQEAYVNINSIPTHIFTWGRWI----EETI-TEKEIVICITGNPGLPGFYTEFAGTLQKELG-DLPVWVIGHA   67
H. sapiens               1   MDSELKEEIPVHEEFILCGGAETQVLKCGPWTDLFHDQSVKRPKLLIFIIPGNPGFSAFYVPFAKALYSLTNRRFPVWTISHA   83
M. musculus              1   MASEVEEQIPVREEFFLCGGVETKIIKCGPWTNLFEKQDVSKPKQLIFIIPGNPGYSAFYVPFAKALYTLMKSRFPVWIISHA   83

D. melanogaster   68   GHDDPPEASIRE-----VPQLSGNEELFNLDGQIRHKIAFIEKYVPSDVKIHLIGHSIGAWMILQLLE-NERIRSRIQKCYMLFPTVERMMESPNGWVF   160
H. sapiens        84   GHALAPKDKKILTTSE-DSNAQEIKDIYGLNGQIEHKLAFLRTHVPKDMKLVLIGHSIGSYFTLQMLKRVPEL--PVIRAFLLFPTIERMSESPNG---   176
M. musculus       84   GFSVTPKDKKVLAAPQEESNAQKIEDVYGLNGQIEHKIAFLRAHVPKDVKLILIGHSVGTYMTLHVMKRVPEL--PVAHAFLLFPTIERMSESPNG---   177
                                                                  HxxxD       GxSxG

D. melanogaster   161   TKVAMPLYSVFGYI--FFS----FFNFLPVWLRLMLIQIYFLIFSIPRQF-LGTALKYSKPSVAEKVVFLADDEMARVRGIQREIVEQNLDLLKFYYGT   252
H. sapiens        177   -RIATPLLCWFRYV--LYVTGYLLLKPCPETIKSLLIRRGLQVMNLENEFSPLNILEPF---CLANAAYLGGQEMMEVVKRDDETIKEHLCKLTFYYGT   269
M. musculus       178   -KFATPFLCQFRYL--LYATSYLLFKPCPEVIKSFIIQKLMGQMNIKLELPLTDILQPF---CLANAAYLGSQEMVQIVKRDDDIIKEFLPKLKFYYGK   270

D. melanogaster   253   TDGWVPISYYDQLKKDYPKVDAQLDTKKIDHAFVLRHSQPMAVIVRDMIQ-QHRRV   307
H. sapiens        270   IDPWCPKEYYEDIKKDFPEGDIRLCEKNIPHAFITHFNQEMADMIADSLKDDLSKM   325
M. musculus       271   TDGWCPVKYYEDMKKDFPEGNIYLCEKGIPHAFVLDFSQEMATIVAEWINNRPPRK   326
                                                      HxxxxD
```

(B)

```
M. musculus (Isoform 1)        1   MASEVEEQIPVREEFFLCGGVETKIIKCGPWTNLFEKQDVSKPKQLIFIIPGNPGYSAFYVPFAKALYTLMKSRFPVWIISHA   83
M. musculus (Isoform 2)        1   MASEVEEQIPVREEFFLCGGVETKIIKCGPWTNLFEKQDVSKPKQLIFIIPGNPGYSAFYVPFAKALYTLMKSRFPVWIISHA   83
M. musculus (Isoform 3)        1   MASEVEEQIPVREEFFLCGGVETKIIKCGPWTNLFEKQDVSKPKQLIFIIPGNPGYSAFYVPFAKALYTLMKSRFPVWIISHA   83

M. musculus (Isoform 1)   84   GFSVTPKDKKVLAAPQEESNAQKIEDVYGLNGQIEHKIAFLRAHVPKDVKLILIGHSVGTYMTLHVMKRVPELPVAHAFLLFPTIERMSESPNG   177
M. musculus (Isoform 2)   84   GFSVTPKDKKVLAAPQEESNAQKIEDVYGLNGQIEHKIAFLRAHVPKDVKLILIGHSVGTYMTLHVMKRVPELPVAHAFLLFPTIERMSESPNG   177
M. musculus (Isoform 3)   84   GFSVTPKDKKVLAAPQEESNAQKIEDVYGLNGQIEHKIAFLRAHVPKDVKLILIGHSVGTYMTLHVMKRVPELPVAHAFLLFPTIERMSESPNG   177

M. musculus (Isoform 1)   178   KFATPFLCQFRYLLYATSYLLFKPCPEVIKSFIIQKLMGQMNIKLELPLTDILQPFCLANAAYLGSQEMVQIVKRDDDIIKEFLPKLKFYYGK   270
M. musculus (Isoform 2)   178   KFATPFLCQFRYLLYATSYLLFKPCPEVIKSFIIQKLMGQMNIKLELPLTDILQPFCLA----------------------------------   270
M. musculus (Isoform 3)   178   KFATPFLCQFRYLLYATSYLLFKPCPEVIKSFIIQKLMGQMNIKLELPLTDILQPFCLANAAYLGSQEMVQIVKRDDDIIKEFLPKVTMGTFW   270

M. musculus (Isoform 1)   271   TDGWCPVKYYEDMKKDFPEGNIYLCEKGIPHAFVLDFSQEMATIVAEWINNRPPRK   326
M. musculus (Isoform 2)   271   --------------------------------------------------------   326
M. musculus (Isoform 3)   271   GS-------------------------------------------------------   326
```

Supplementary Figure 4. Alignment of drosophila, human and mouse LDAH orthologs, and predicted catalytic sites. (A) Sequence alignment of drosophila, mouse and human LDAH isoform 1. The predicted esterase/lipase motif (GxSxG) and two potential acyltransferase motifs (HxxxD and HxxxxD) are highlighted. **(B)** Sequence alignment of mouse LDAH isoforms generated by alternative splicing. The potential catalytic sites are highlighted, and the C-terminal amino acids that differ between isoforms 3 and 1 are bolded.

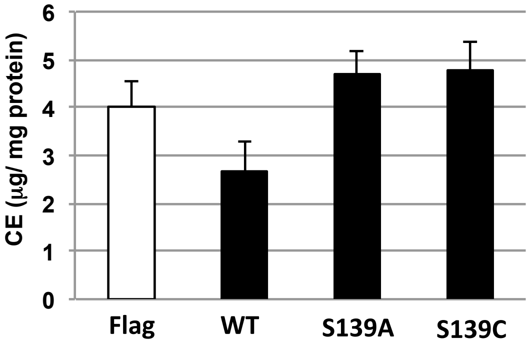

Supplementary Figure 5. Mutation in GXSXG motif abolishes LDAH's effect on CE stores. Flag, Flag-LDAH WT, Flag-LDAH S139A, or Flag-LDAH S139C were transfected to HEK293 cells, and cells were treated with cholesterol-methyl-β-cyclodextrin (CHOL:MβCD; 10 µg/mL) for 24 h. Cholesterol ester (CE) levels were calculated by subtracting free cholesterol from total cholesterol.

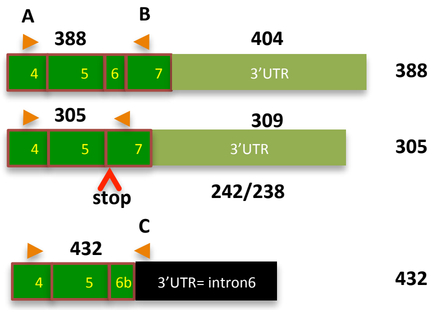

primer sequences
A: E4-F1-5'-TTCACGTGATGAAGCGAGTC-3'
B: E7-R1-5'-GGACACCAGCCATCAGTTTT-3'
C: I6-RI -5'-CCATTCTTGCCTTGTGTCCT-3'

Supplementary Figure 6. Location and sequences of primers to identify LDAH isoforms 1, 2, and 3.

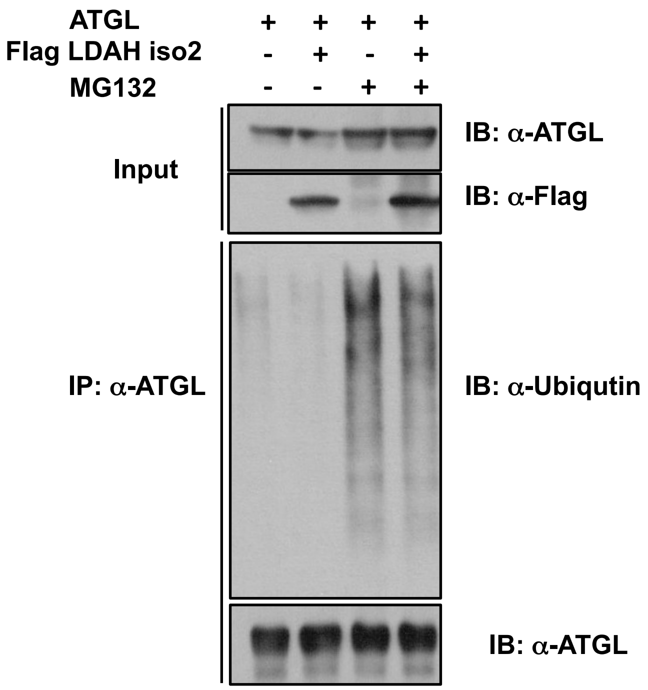

Supplementary Figure7. LDAH isoform 2 does not affect ubiquitination of ATGL. ATGL and/or Flag-LDAH isoform 2 were transfected to HEK293 cells as indicated. 24 h after transfection cells were treated with OA (360 μM for 24 h) followed by MG132 for 8 h as indicated in the figure. ATGL was pulled-down with an anti-ATGL antibody and protein A-agarose. Immunoblots were performed with anti-ubiquitin, anti-Flag, and anti-ATGL antibodies.

2. Full-length unprocessed blots included in main figures:

Figure 3A, anti-mouse LDAH

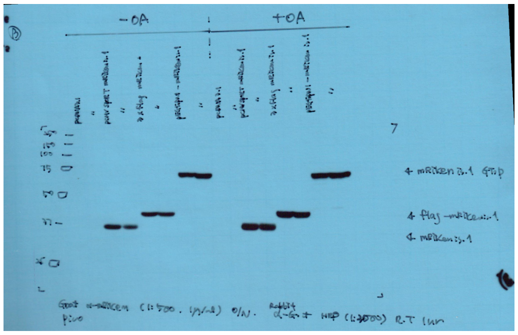

Figure 3B, anti-human LDAH

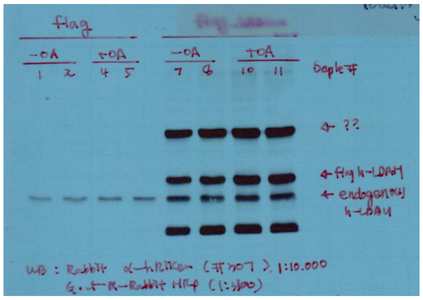

Figure 3B, reblot with anti Tubulin

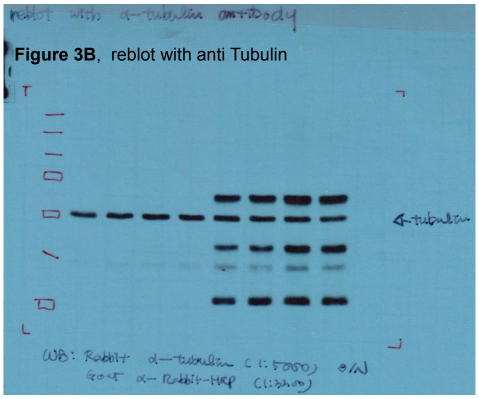

Figure 3C, anti-human LDAH

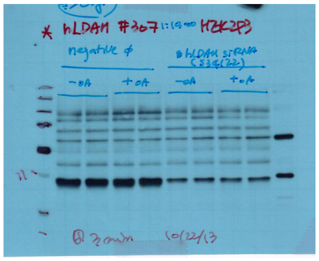

Figure 3C, reblot **with** anti-Tubulin

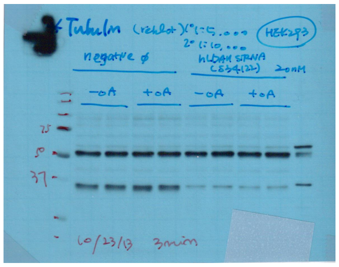

Figure 3D Top, anti-mouse LDAH

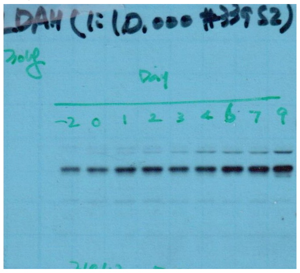

Figure 3D middle, anti-PLIN1

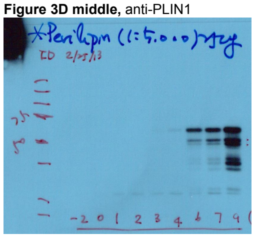

Figure 3D bottom anti-GAPDH

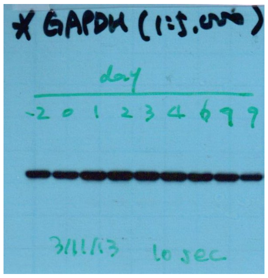

Figure 3E (anti-mouse LDAH (left) and reprobe with anti-Actin (right)

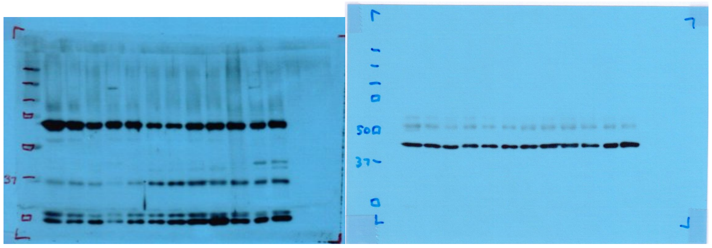

Figure 4B, anti-Flag-HRP

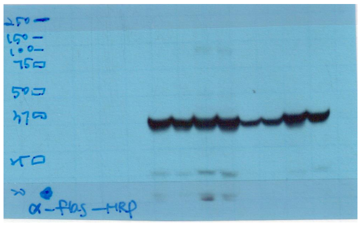

Figure 4D, acrylamide gel staining

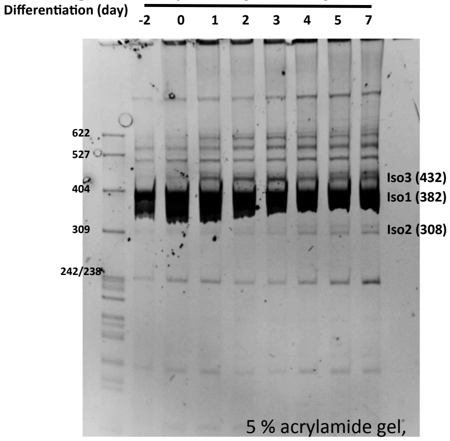

Figure 4E, anti-Flag-HRP

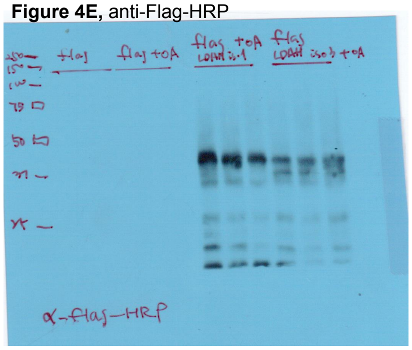

Figure 4F, anti-Flag-HRP (Top), ponseau staining (Bottom)

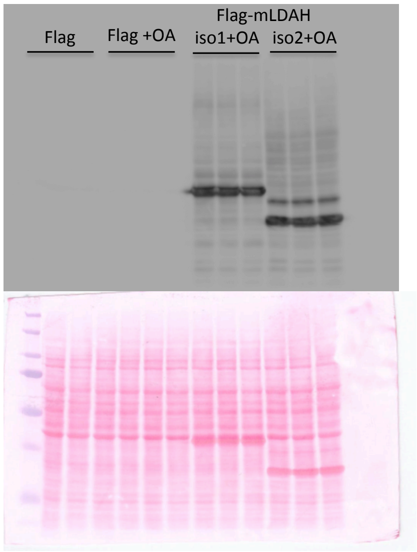

Figure 6A, anti-ATGL, anti-Flag, and anti-Actin

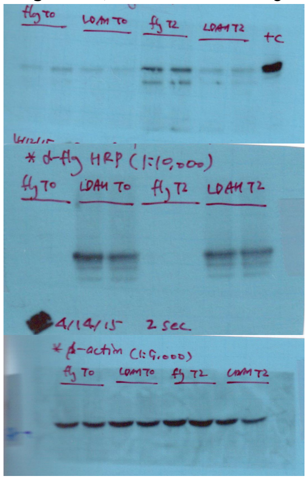

Figure 6B, anti-ATGL

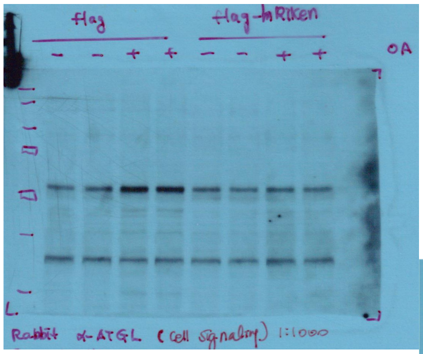

Figure 6C, anti-Flag-HRP

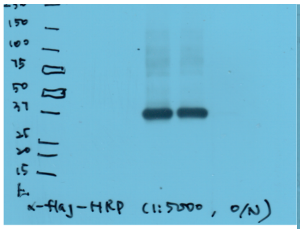

Figure 6C, anti-ATGL

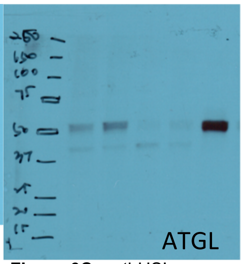

Figure 6C, anti-PLIN1

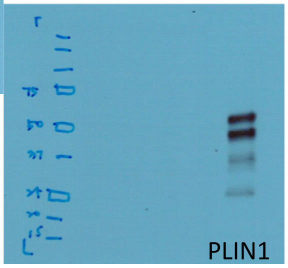

Figure 6C, anti-HSL

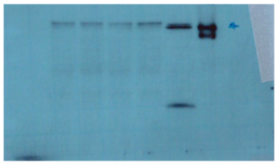

Figure 6C, anti-PLIN3

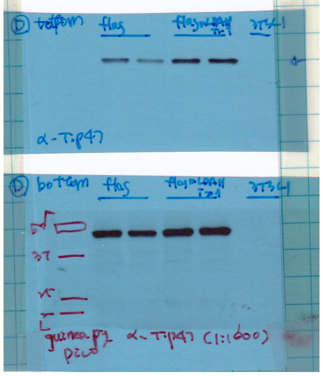

Figure 6C, anti-PLIN2

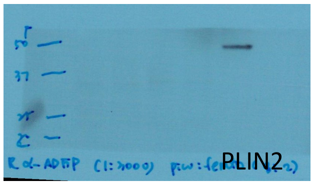

Figure 6C, anti-Actin

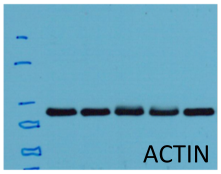

Figure 6D, anti-human LDAH (left) ATGL (right)

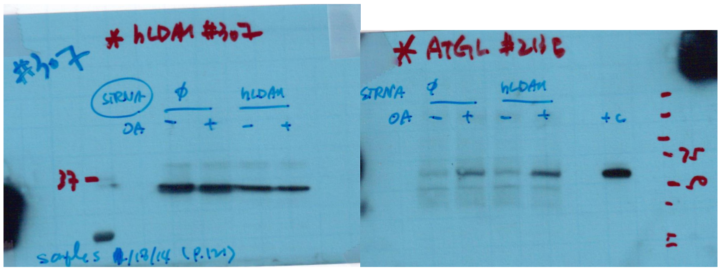

Figure 6E, anti-LDAH

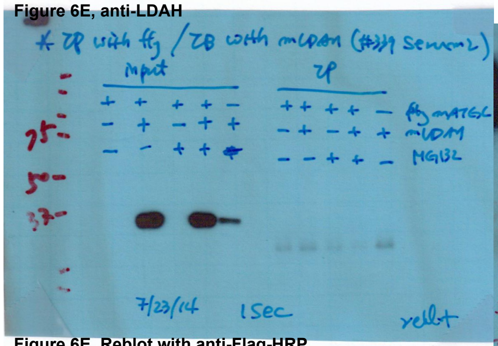

Figure 6E, anti-Ubiquitin

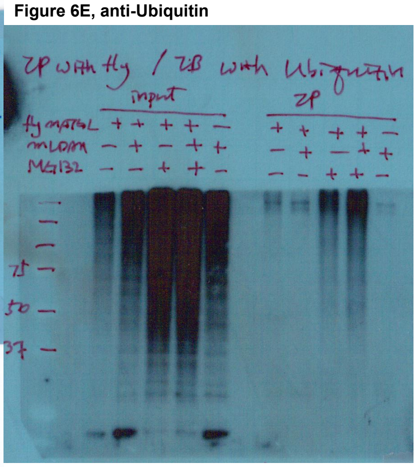

Figure 6E, Reblot with anti-Flag-HRP

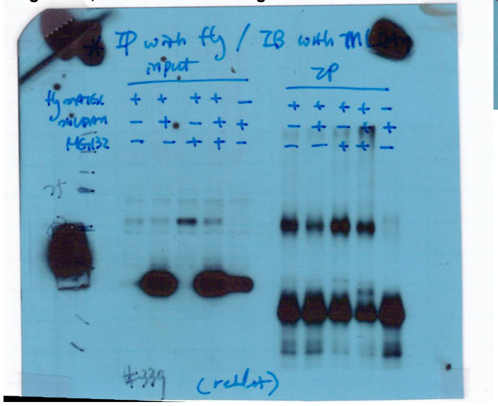

Supplement: Supplementary file 1 — Supplementary material [file 41598_2017_2963_MOESM1_ESM.pdf]
